# Causal links between blood inflammation markers and postherpetic neuralgia risk: insights from a two-sample Mendelian randomization study

**DOI:** 10.3389/fneur.2024.1411541

**Published:** 2024-09-25

**Authors:** Yu Wang, Tian Jia

**Affiliations:** ^1^Department of Pharmacy, Northwest University First Hospital, Xi’an, Shaanxi, China; ^2^Department of Child Healthcare, Northwest University First Hospital, Xi’an, Shaanxi, China

**Keywords:** Mendelian randomization, postherpetic neuralgia, blood inflammation, genetic variants, MIP1β, IL-10, IL-12p70

## Abstract

**Introduction:**

Previous studies have suggested an association between blood inflammation-related factors and postherpetic neuralgia. However, the causal relationship between blood inflammation-related factors and postherpetic neuralgia remains unclear.

**Methods:**

We employed a bidirectional Two-sample Mendelian randomization (MR) analysis to explore the causal relationship between blood inflammation-related factors and postherpetic neuralgia. The instrumental variables were obtained from a large Genome-wide association study (GWAS) meta-analysis dataset of European descent. The instrumental variables of the blood inflammation-related factors come from the database numbers GCST004420 to GCST004460 and GCST90029070. Postherpetic neuralgia has 195,191 samples with a total of 16,380,406 single nucleotide polymorphisms (SNPs). MR analyses were performed using inverse-variance weighted, MR-Egger, and weighted median methods.

**Results:**

The MR results revealed a significant causal effect of Macrophage Inflammatory Protein 1 Beta (MIP1β) on reducing the risk of postherpetic neuralgia (95%CI = 0.492–0.991, *p* = 0.044). Additionally, higher levels of interleukin (IL)-10 (95%CI = 0.973–0.998, *p* = 0.019) and IL-12p70 (95%CI = 0.973–0.997, *p* = 0.013) were associated with a lower risk of postherpetic neuralgia. Other inflammatory markers showed no significant causal relationship with this condition.

**Conclusion:**

This study identifies MIP1β, IL-10, and IL-12p70 as potential therapeutic targets for preventing or treating postherpetic neuralgia, underscoring the need for further research in this area.

## 1 Introduction

Postherpetic neuralgia is a frequent complication of herpes zoster, characterized by pain persisting for over 3 months following the onset of herpes zoster (1, 2). This intense neuropathic pain is distinguished by ongoing discomfort in the affected dermatome, leading to significant disruptions in patients’ overall well-being, including disturbances in sleep, feelings of depression and anxiety, as well as a reduction in physical function. In severe cases, the pain can be incapacitating or even life-threatening (2, 3). Consequently, postherpetic neuralgia can result in substantial physical, emotional, functional, and social limitations, rendering it the most severe aftermath of herpes zoster.

The pathogenesis of postherpetic neuralgia is multifactorial and complex. One possible mechanism of postherpetic neuralgia is immune activation, in which the immune system produces cytokines and other inflammatory molecules that damage nerve cells and cause persistent pain (4). Furthermore, chronic inflammation can alter the way the central nervous system processes pain signals such that pain persists even after the initial injury has healed (5). Immune-mediated inflammation is a key factor in the development of neuropathic pain. Blood inflammation-related factors are thought to be involved in neuropathic pain. C-reactive protein (CRP) is a well-known biomarker of systemic inflammation and previous observational studies have shown a positive correlation between its levels and the pain in patients with advanced cancer (6) and postherpetic neuralgia (7, 8). Several observational studies have found that serum interleukin (IL)-6 and IL-10 levels are higher in patients with herpes zoster compared with healthy controls (9, 10), suggesting an association of IL-6 and IL-10 and the development of postherpetic neuralgia. IL-10 is considered an anti-inflammatory marker that inhibits the synthesis of pro-inflammatory cytokines (11, 12). However, traditional observational studies have some limitations, and the associations between inflammatory markers and postherpetic neuralgia reported in previous studies may still be explained by reverse causality and residual confounding. Therefore, whether there is a causal relationship between inflammatory factors and postherpetic neuralgia remains uncertain.

To establish a cause-and-effect link between inflammatory factors and the development of postherpetic neuralgia, we can employ Mendelian randomization (MR). MR is a research design that uses genetic variants as instrumental variables to estimate the causal impact of risk factors on health outcomes (13, 14). In contrast to conventional multivariable observational analyses, MR is less susceptible to the influence of confounding variables and measurement errors, and it avoids biases stemming from reverse causation (15). Consequently, MR has emerged as a reliable method for obtaining robust estimates of the causal influence of various risk factors on health outcomes, often yielding findings similar to those obtained from randomized controlled trials when available (16).

In the present study, we employed a bidirectional MR analysis using genetic variations as instrumental variables to assess the causal relationship between blood inflammation-related factors and postherpetic neuralgia in both the forward and reverse directions. We found no evidence of a link between genetically predicted inflammatory variables and levels of potential confounders. Thus, by assuming that the connection between genetic variants and postherpetic neuralgia exclusively operates through exposure, MR analysis can be employed to ascertain the causal influence of inflammatory factors on the risk of developing postherpetic neuralgia.

## 2 Materials and methods

### 2.1 Study design

Study the causal relationship between blood inflammation-related factors [Cutaneous T-cell Attracting Chemokine (CTACK), Beta Nerve Growth Factor (*β*NGF), Vascular Endothelial Growth Factor (VEGF), Macrophage Migration Inhibitory Factor (MIF), Tumor Necrosis Factor (TNF)β, TNFα, TNF-Related Apoptosis-Inducing Ligand (TRAIL), Stromal Cell-Derived Factor 1 Alpha (SDF1α), Stem Cell Growth Factor Beta (SCGFβ), Stem Cell Factor (SCF), IL-16, Regulated upon Activation, Normal T cell Expressed and Secreted (RANTES), Platelet-Derived Growth Factor BB (PDGFbb), Macrophage Inflammatory Protein 1 (MIP1)*β*, MIP1α, Monokine Induced by Gamma Interferon (MIG), Macrophage Colony-Stimulating Factor (MCSF), Monocyte Chemoattractant Protein (MCP)3, MCP1, IL-12p70, Interferon Gamma-Induced Protein 10 (IP10), IL-18, IL-17, IL-13, IL-10, IL-8, IL-6, IL1ra, IL-1β, Hepatocyte Growth Factor (HGF), IL-9, IL-7, IL-5, IL-4, IL2rα, IL-2, Interferon Gamma (IFN-*γ*), Growth-Regulated Oncogene Alpha (GROα), Granulocyte Colony-Stimulating Factor (GCSF), Basic Fibroblast Growth Factor (bFGF), Eotaxin, CRP] (17, 18) and postzoster neuralgia (19). The MR randomization is based on three main assumptions: (1) The selected genetic variant single nucleotide polymorphisms (SNPs) should be closely associated with the exposure; (2) The selected genetic variant SNPs are independent of the outcome through confounding factors; and (3) The selected SNPs affect the outcome through the exposure, but not directly related.

### 2.2 Data sources

Data in the two-sample MR analysis were obtained from the Genome-Wide Association Study (GWAS) database.^1^ The samples of blood inflammation-related factors and postherpetic neuralgia are all from Europe: the instrumental variables of the blood inflammation-related factors come from the database numbers GCST004420 to GCST004460 and GCST90029070 (17, 18). Postherpetic neuralgia has 195,191 samples with a total of 16,380,406 SNPs (19).

### 2.3 Analytical method

MR analysis was performed using the R programming language TwoSampleMR version 0.5.7. A strict *p*-value threshold (*p* < 5 × 10–8) was used to select SNPs as instrumental variables to evaluate the causal effect of blood inflammation-related factors and postherpetic neuralgia. To address the issue of significant linkage disequilibrium (LD), clustering with *R*^2^ < 0.001 and a window size of 10,000 kb was performed using data from the European reference panel of 1,000 genomes (20). This study used inverse-variance weighted (IVW), MR Egger, and Weighted median methods. The MR Egger regression intercept was used to evaluate pleiotropy, and when *p* > 0.05, it was considered that there was no pleiotropy. Heterogeneity was tested using Cochrane’s Q statistic, and Q_pval >0.05 was considered to have no heterogeneity. Positive results at least ensure that IVW is significant (*p* < 0.05), and the *β* directions of IVW, MR Egger, and Weighted median are consistent.

### 2.4 Statistical analyses

This study used the IVW, weighted median, MR-Egger, and Weighted median methods to investigate the causal relationships between blood inflammation-related factors and postherpetic neuralgia. The MR Egger regression intercept was utilized to assess pleiotropy, and if *p* > 0.05, it was determined that there was no evidence of pleiotropy. Heterogeneity was evaluated using Cochrane’s Q statistic, and Q_pval >0.05 was indicative of no significant heterogeneity. Positive results ensured that IVW was statistically significant (*p* < 0.05), and that the *β* directions of IVW, MR Egger, and Weighted median were consistent.

## 3 Results

### 3.1 Causal effects of blood inflammation-related factors on postherpetic neuralgia

**Figure 1:**
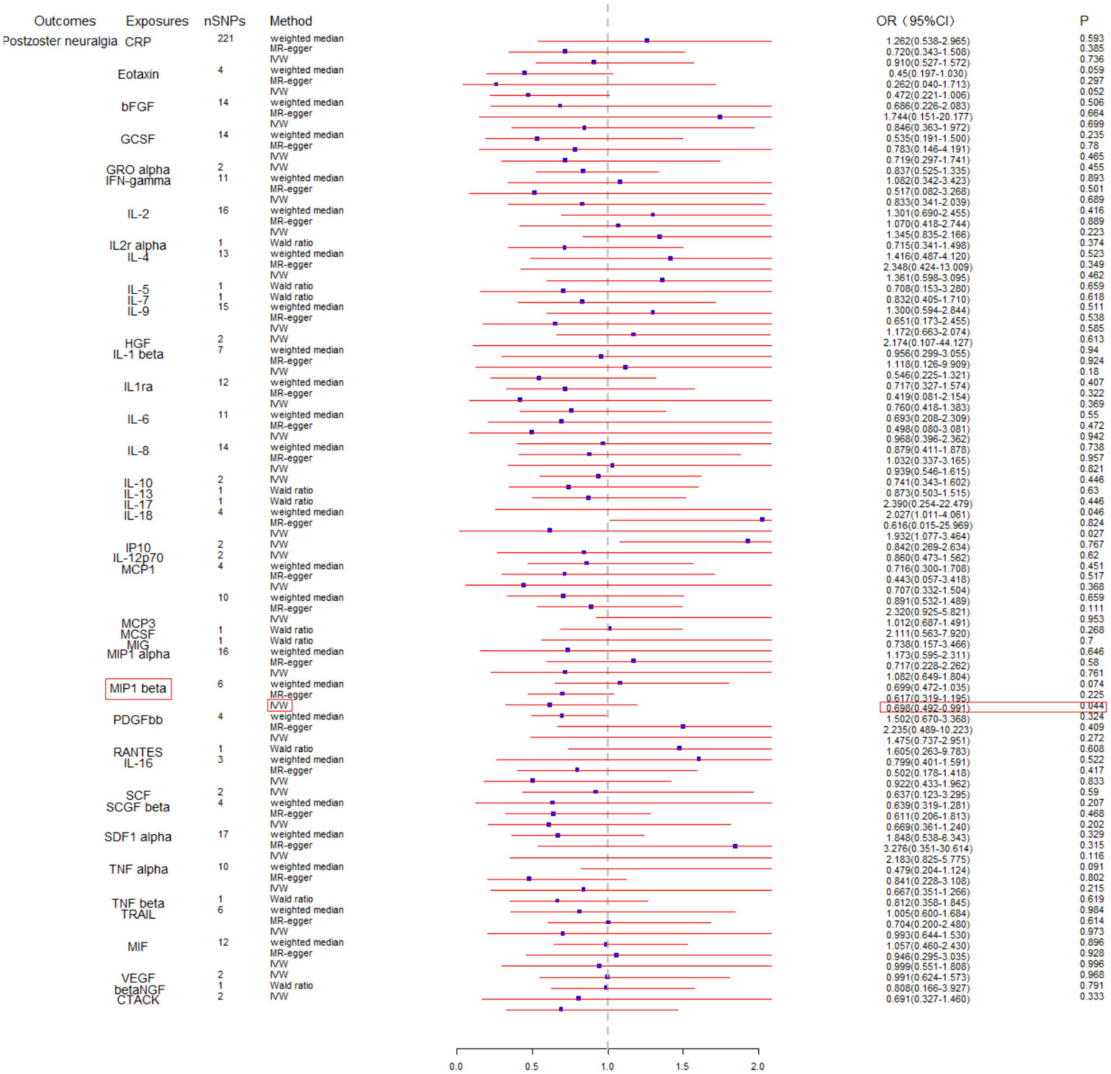
Mendelian randomization analysis of blood inflammation-related factors on postherpetic neuralgia. SNPs, single nucleotide polymorphisms; IVW, inverse-variance weighted.

**Figure 2:**
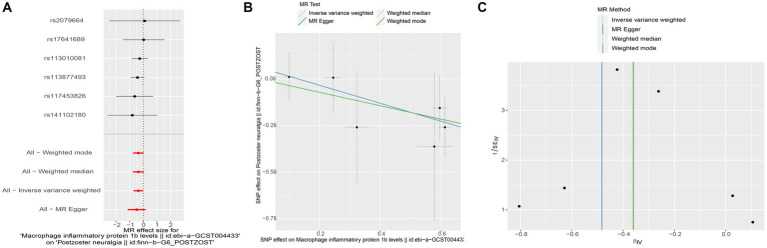
Mendelian randomization analysis of MIP1β associated SNPs on the risk of postherpetic neuralgia. **(A)** Forest plot and **(B)** scatter plot of the potential effects of MIP1β associated SNPs on postherpetic neuralgia. **(C)** Funnel plot of the causal effect of MIP1β associated SNPs on postherpetic neuralgia. MIP1β, Macrophage Inflammatory Protein 1 Beta; SNPs, single nucleotide polymorphisms.

To assess the potential causal impact of blood inflammation-related factors on postherpetic neuralgia, we first examined the causal relationship between blood inflammation-related factors on postherpetic neuralgia by a two-sample MR analysis. We selected blood inflammatory factors (including CTACK, βNGF, VEGF, MIF, TRAIL, TNFβ, TNFα, SDF1α, SCGFβ, SCF, IL-16, RANTES, PDGFbb, MIP1β, MIP1α, MIG, MCSF, MCP3, MCP1, IL-12p70, IP10, IL-18, IL-17, IL-13, IL-10, IL-8, IL-6, IL1ra, IL-1β, HGF, IL-9, IL-7, IL-5, IL-4, IL2rα, IL-2, IFN-*γ*, GROα, GCSF, bFGF, Eotaxin, CRP) as exposures and assessed their impact on the risk of developing postherpetic neuralgia (outcome) in a two-sample MR analysis. The results of IVW model analysis showed that there was a statistically significant inverse relationship between MIP1β levels and postherpetic neuralgia (95% CI = 0.492 to 0.991, *p* = 0.044) (Figure 1). In addition, no other factors have been observed to have an obvious causal relationship with postherpetic neuralgia (Figure 1). We further subjected the 6 SNPs associated with MIP1β to MR analysis. Scatter plots and forest plots also showed a statistically significant association between genetic susceptibility to MIP1β levels and postherpetic neuralgia (Figures 2A,B). Funnel plots also did not observe evidence of heterogeneity in effect estimates between variants (Figure 2C). The results suggest that an elevation of MIP1β reduces the risk of postherpetic neuralgia.

### 3.2 Causal effects of postherpetic neuralgia on blood inflammation-related factors

**Figure 3:**
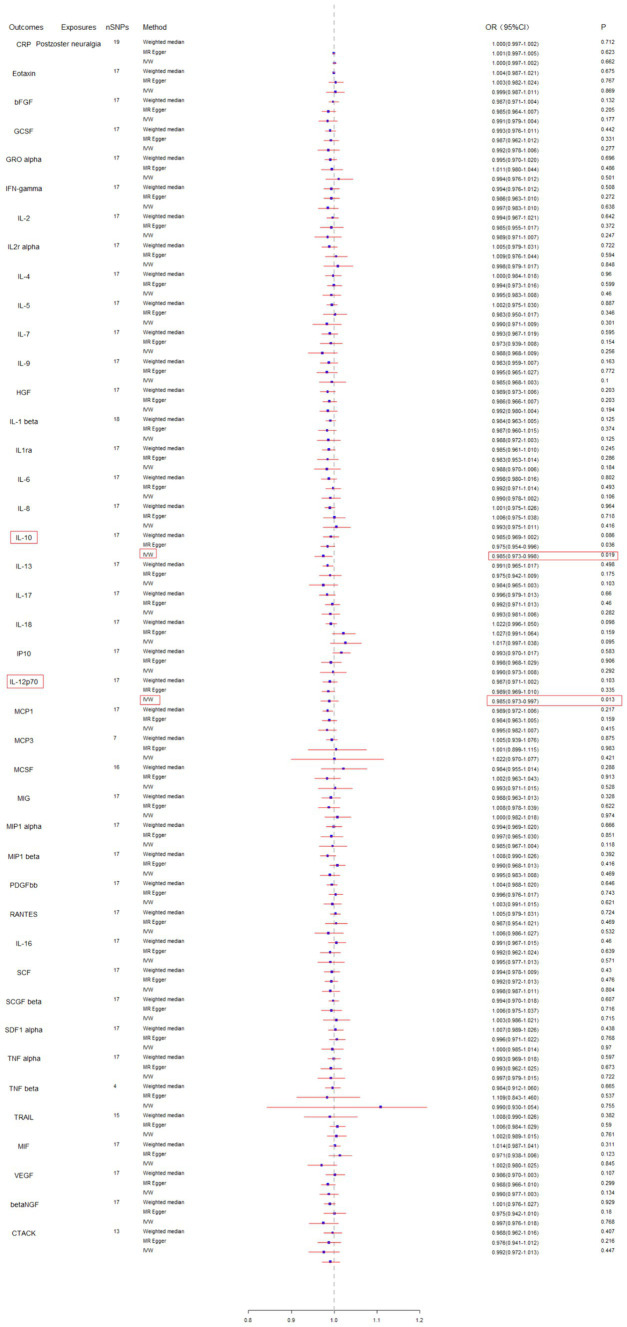
Mendelian randomization analysis of postherpetic neuralgia on the levels of blood inflammation-related factors. SNPs, single nucleotide polymorphisms; IVW, inverse-variance weighted.

**Figure 4:**
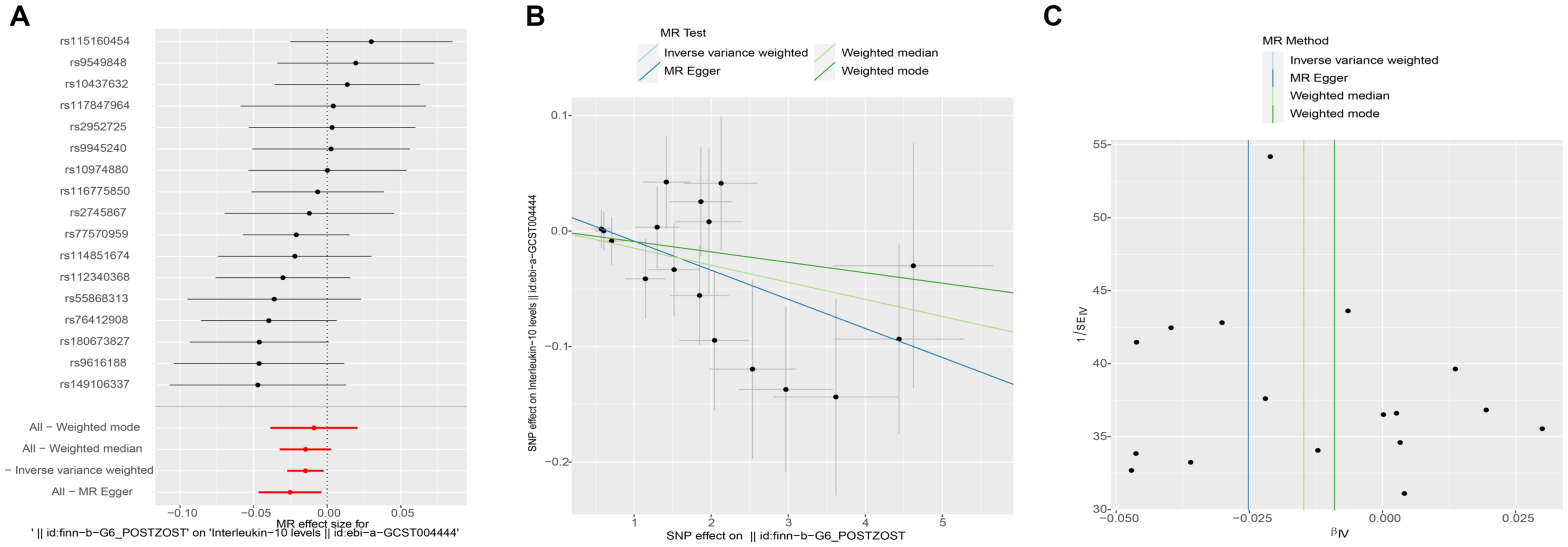
Mendelian randomization analysis of postherpetic neuralgia on interleukin (IL)-10 associated single nucleotide polymorphisms (SNPs). **(A)** Forest plot, **(B)** scatter plot, and **(C)** funnel plot of the causal effect postherpetic neuralgia on IL-10 levels.

**Figure 5:**
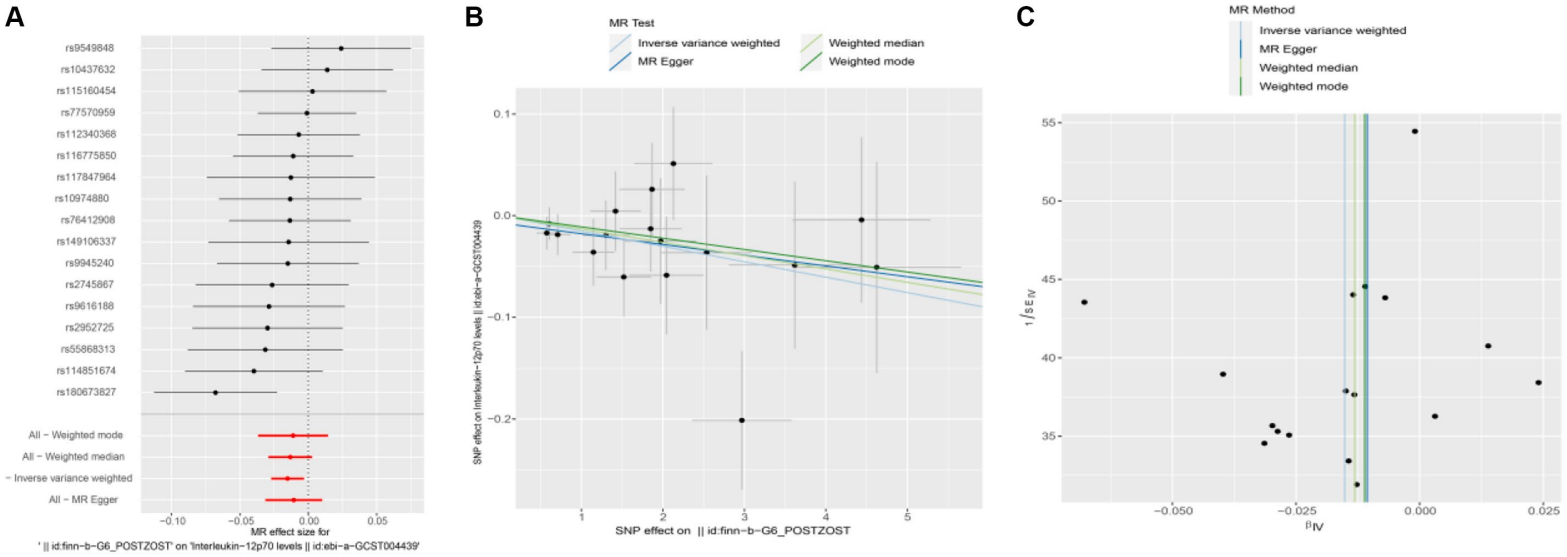
Mendelian randomization analysis of postherpetic neuralgia on interleukin (IL)-12 p70 associated single nucleotide polymorphisms (SNPs). **(A)** Forest plot, **(B)** scatter plot, and **(C)** funnel plot of the causal effect postherpetic neuralgia on IL-12 p70 levels.

Subsequently, we conducted a reverse MR analysis to investigate the causal relationship between postherpetic neuralgia and blood inflammation-related factors. Postherpetic neuralgia was treated as the exposure, and blood inflammatory factors were the outcome. The results of IVW model analysis showed a significant causal relationship between postherpetic neuralgia and decreased levels of IL-10 (95% CI = 0.973–0.998, *p* = 0.019) and IL-12p70 (95% CI = 0.973–0.997, *p* = 0.013) (Figures 3, 4A,B, 5A,B), and both factors have no heterogeneity or pleiotropic effects (Figures 4C, 5C). In addition, no obvious causal relationship was found with blood inflammation-related factors other than IL-10 and IL-12p70. These results suggest that lower levels of IL-10 and IL-12p70 may be a consequence of postherpetic neuralgia, which could exacerbate the inflammatory response and pain.

## 4 Discussion

Postherpetic neuralgia is a common complication of herpes zoster that can lead to severe physical, psychological, functional, and social impairment, making it the most debilitating sequelae of shingles (21). In the present study, we explored the causal relationship between blood inflammation-related factors and postherpetic neuralgia using a bidirectional, two-sample MR method. We obtained the following results: (1) There is a causal relationship between MIP1β and postherpetic neuralgia; As MIP1β increases, the risk of postherpetic neuralgia decreases without heterogeneity or pleiotropic effects. (2) There is a causal relationship between IL-10 and IL-12p70 and postherpetic neuralgia; Increasing in IL-10 and IL-12p70 can reduce the risk of postherpetic neuralgia, and both factors are not heterogeneous and pleiotropic.

Macrophage inflammatory protein 1 beta (MIP1β), also known as CCL4 (C-C motif chemokine ligand 4), is a cytokine involved in the immune response (22). It plays a crucial role in recruiting immune cells to sites of infection or inflammation. Elevated level of MIP1β has been observed in conditions associated with chronic pain, such as neuropathic pain (23, 24), inflammatory disorders (25), and certain types of arthritis (26). We here reported that there is a negative relationship between levels of MIP1β and postherpetic neuralgia. As the increase of MIP1β, the risk of postherpetic neuralgia decreases. The relationship between MIP1β and pain is complex and can vary depending on the specific context and underlying condition. Further research is needed to fully understand the mechanisms through which MIP1β contributes to pain and to explore its potential as a target for pain management strategies.

Cytokines are pleiotropic small proteins that are key players in the induction and maintenance of neuropathic pain. In previous studies examining the relationship between inflammatory factors and pain, it was possible to evaluate the most biomarkers, mainly IL-6, IL-2, and IL-10 (27–29). IL-10 is recognized as an anti-inflammatory cytokine with analgesic properties (30). Fukuyasu et al. found that serum IL-10 level is an objective biomarker of the severity of herpes zoster, and that the serum IL-10 level can be a practical predictor of the duration of neuralgia (9). The application of IL-10 reduces pain behavior in different kinds of pain models (31). Wang et al. found that patients with mild lumbar radicular pain had lower serum IL-10 levels than those with severe lumbar radicular pain (32). Here, we found that increasing in IL-10 reduces the risk of postherpetic neuralgia. These findings suggest that IL-10 may be a therapeutic target for postherpetic neuralgia.

Interleukin-12p70 (IL-12p70) is primarily known for its role in the immune system, particularly in the activation of immune cells involved in fighting infections. However, there is emerging research suggesting that IL-12p70 may also play a role in pain modulation. Rahmawati et al. reported that serum IL-12p70 levels increased in women with endometriosis compared to the control group (33). Singh et al. found that IL-12p70 changed significantly more in Western Ontario McMaster Osteoarthritis Index pain responders compared to pain non-responders (34). However, in our present study, elevated levels of IL-12p70 reduces the risk of postherpetic neuralgia. The relationship between IL-12p70 and pain is complex and multifaceted. Further research is needed to fully understand the role of IL-12p70 in postherpetic neuralgia processing and to explore its potential as a target for pain management strategies.

This study has its own set of limitations: (1) The GWAS data are derived exclusively from European populations. Consequently, the findings may not be extrapolated to other races and regions. More comprehensive studies are warranted among diverse ethnic groups; (2) Despite leveraging the largest available large-scale GWAS data, subsequent research endeavors should focus on further augmenting the sample size to yield more precise assessments.

In summary, we used a two-sample Mendelian randomization method to explore the causal relationship between blood inflammation-related factors and postherpetic neuralgia. This study found that as MIP1β increases, the risk of postherpetic neuralgia decreases, suggesting a negative relationship between MIP1β and postherpetic neuralgia. While increases in IL-10 and IL-12p70 can reduce the risk of postherpetic neuralgia. These findings provide new evidence for the treatment and prevention of postherpetic neuralgia.

## Data Availability

The datasets presented in this study can be found in online repositories. The names of the repository/repositories and accession number(s) can be found in the article/supplementary material.

## Footnotes

^1^ https://gwas.mrcieu.ac.uk/
